# Simulating the Transmission of Rift Valley Fever Virus via Raw Milk Consumption in Inter-Epidemic Periods

**DOI:** 10.3390/v18080889

**Published:** 2026-08-13

**Authors:** Esra Buyukcangaz, Amna Tariq, Victor Hugo Peña-García, Donal Bisanzio, Koree French, Brian E. Dawes, Angelle Desiree LaBeaud

**Affiliations:** 1 Department of Pediatrics, Division of Infectious Diseases, Stanford University, Stanford, CA 94305, USA; atariq1@stanford.edu (A.T.); victorhugopega@gmail.com (V.H.P.-G.); doctorfrench2021@gmail.com (K.F.); dlabeaud@stanford.edu (A.D.L.); 2 Department of Veterinary Sciences, University of Turin, largo Paolo Braccini 2, Grugliasco, 10095 Turin, Italy; donal.bisanzio@gmail.com; 3 Department of Medicine, Division of Infectious Diseases and Geographic Medicine, Stanford University, Stanford, CA 94305, USA; bedawes@stanford.edu

**Keywords:** milk transmission, one health, Rift Valley Fever Virus (RVFV), stochastic simulation

## Abstract

Rift Valley fever virus (RVFV) is a zoonotic, vector-borne disease affecting livestock and humans. The consumption of raw unpasteurized milk has been epidemiologically linked to RVFV exposure in humans and recognized as a potentially important non-vector route for RVFV transmission. This study aims to investigate the potential of RVFV transmission to humans through the consumption of infected raw milk. For this purpose, we conducted agent-based stochastic simulations to assess the potential for RVFV transmission through ingestion of contaminated raw milk. We created synthetic populations of cows, humans, and mosquitoes representing local farm dynamics in Kisumu, Kenya. A total of 1000 farms were simulated with binomial and Poisson distributions utilized to generate cow and human populations, respectively. Given our prior field data, we varied the probability of consuming raw milk from 1% to 80%. We also varied the probability of infectious raw milk causing RVFV infection at 10% and 40%. Results indicate that when the probability of infectious raw milk causing an infection is set at 10%, the cumulative incidence of RVFV infections in humans through raw milk consumption varies between 0.4 and 13% during the inter-epidemic periods. When the probability of infectious raw milk causing an infection is set at 40%, the percentage of cumulative milk infections ranges from 3.7 to 28%. Findings highlight the great potential for RVFV infection and spread through infectious milk consumption and the need for further evaluation of milk as a potential pathway of RVFV transmission to humans. Raw milk consumption in RVFV endemic settings remains an ongoing public health threat.

## 1. Introduction

Rift Valley fever (RVF) is a mosquito-borne viral disease caused by a Phlebovirus from the *Phenuiviridae* family, resulting in acute, zoonotic febrile illness in wild animals and domestic ruminants, including cattle, sheep, goats, and camels, as well as humans [1,2,3,4,5,6,7,8]. Transmission can occur by mosquito bites or direct contact with infected animal body fluids or tissues [3,5,7,9]. Infection with Rift Valley fever virus (RVFV) can result in “abortion storms” in pregnant livestock and elevated mortality rates in young animals [3,5]. RVFV can cause severe complications in humans such as encephalitis, abortions, retinitis, hepatitis, and hemorrhagic fever [9,10,11,12,13,14]. RVFV not only inflicts substantial economic repercussions through its direct and indirect effects on animal production during outbreaks [15,16], but it also remains a critical public health issue due to the severity of the illness and lack of approved vaccines or treatments for humans [7,8,11].

RVFV is endemic to Africa in both humans and animals [3,8,11,17,18,19,20,21,22], and was first reported in 1931 [23]. The spread of RVFV to the Indian Ocean and the Arabian Peninsula highlights the potential for further viral dissemination through changes in the environment, global travel, trade, and extensive vector competence [6,24,25,26]. Several transmission pathways of RVFV to humans have been documented, all of which contribute to the overall epidemiological scenario in various ways [9,10,11,13,16,17,18,27,28]. The transmission pathways include direct exposure to contaminated animal tissues, blood, or body fluids, inhalation of aerosolized infected fluids, and transmission by bites via infected mosquito vectors [5,8]. In Africa, there is a notable prevalence of RVFV circulation among domestic ruminants [20,22,29,30,31]. These findings underscore cattle as a significant amplifying host and a potential source of human exposure via standard husbandry and milk-handling methods. The consumption of raw unpasteurized milk has been epidemiologically linked to RVFV exposure in humans and recognized as a potentially important non-vector route for RVFV transmission [4,10,18,32,33,34,35,36,37,38]. Dawes et al. [39] evaluated the viability of RVFV in milk by measuring RNA and infectious viral titers over time at different temperatures. They demonstrated that RVFV can remain potentially infectious for at least 4 days in refrigerated milk and up to 2 days in milk stored at temperatures below ambient. [39]. Commonly performed pasteurization techniques and boiling of milk effectively inactivate RVFV [39]. In numerous retrospective research studies, unpasteurized milk has been identified as a potential risk factor for RVFV infection [18,31,32,33,34,35,37,38,40,41]. A study determined that the consumption of raw animal milk was linked to an approximately threefold increase in odds of RVFV seropositivity in humans [41]. During inter-epidemic periods, individuals in Kenya and Tanzania who consume raw milk and participate in milking activities exhibit markedly elevated odds of RVFV seropositivity, suggesting that milk-related exposure may facilitate undetected transmission during these periods and potentially represents a public health risk due to foodborne and occupational exposure pathways [18,32,35]. In 2022, an early warning system alert reported Western Kenya to be at risk of becoming a new RVFV hotspot, underscoring the need to investigate all potential RVFV transmission pathways, including raw milk consumption in this region [32,42,43]. Therefore, our team has undertaken preliminary studies on RVFV epidemiology in Kenya, where cattle husbandry constitutes a significant industry [19,31,38,41,43]. We aim to investigate the transmission of milk in this setting.

Despite being supported by certain studies [18,37,40,44], the transmission dynamics of RVFV during inter-epidemic periods are poorly understood. The understanding of RVFV inter-epidemic persistence now recognizes a variety of systems ranging from low-level persistence in endemic/epidemic systems to continuous hyperendemicity [45]. These systems may be maintained by transovarial transmission in floodwater *Aedes* spp., transmission in livestock, and/or transmission in wildlife or combinations of these scenarios. In endemic/epidemic systems, RVFV is thought to persist through transovarial transmission and sporadic mammalian infections, whereas in hyperendemic settings continuous mosquito–mammalian transmission is sufficient for maintenance.

However, the specific involvement of these wild mammal species in the transmission and, in particular, the maintenance of the virus remains unclear. Numerous studies have substantiated RVFV infection in African buffalo (*Syncerus caffer*) [18,46,47], which may be important to the RVFV enzootic cycle in some regions due to their dependence on water and their proximity to livestock and humans. Water accessibility is a critical ecological component that promotes mosquito growth and reproduction [48,49]. The *Aedes* genus’ biological habitats are predominantly associated with transient water bodies, like flooded areas, temporary ponds, puddles, and rice fields [37,50]. Additionally, the transition to epidemic transmission is strongly associated with increased water sources for mosquito reproduction, such as flooding from increased rainfall, irrigation, and dams [18,51].

According to Gerken et al. [40] behavioral factors, in addition to environmental factors, can affect the likelihood of RVFV inter-epidemic transmission. The odds of RVFV infection were substantially higher in men than in women, and exposure to the virus was significantly increased by animal contact, butchering, milking, and handling aborted material [40] and renewal of animal herds, exchanging practices [52,53], and nomadic husbandry of animals [44,54]. In inter-epidemic periods, the Rift Valley fever virus (RVFV) may endure in cattle populations, posing a risk of infection to humans by contact with aborted materials or unpasteurized milk. Retrospective analyses of archived human samples have repeatedly yielded evidence of inter-epidemic transmission [18,44,55]. Undetected infections, especially in cattle, serve as a significant reservoir for recurring epidemics [16,56].

Transmission models are essential tools for elucidating complicated structures, particularly mechanistic models, which are well-suited for data-limited contexts [57]. The emergence of such models in the field of infectious disease epidemiology may reflect a growing confidence in mechanistic models [58,59,60]. Mathematical models of RVFV transmission have largely focused on vector-mediated dynamics, with predominantly concentrated fundamental frameworks developed by Gaff et al. [61] utilizing Susceptible–Exposed–Infected (SEI) models for *Aedes* and *Culex* mosquito populations, combined with a Susceptible–Exposed–Infected–Recovery (SEIR) model for livestock [57]. Later, models were extended to incorporate humans as spillover hosts infected from animals [62], and modified to capture regional connectivity and population mobility [57,59,60,61,63]. Consequently, the relative contribution of animal health, milk production, pooling, transport, and consumption to human RVFV risk remains poorly quantified. Although it has been hypothesized and there is overwhelming epidemiologic evidence [32,53], the experimental direct transmission of RVFV among mammals via milk has never been directly demonstrated. Furthermore, direct transmission amplifies the potential outbreaks, exposing densely populated livestock areas at a high risk of infection [58]. Milk transmission may also pose a risk to those in urban areas without direct animal contact [64]. Despite these improvements, existing models typically neglect milk-related exposure pathways, underscoring a significant deficiency in current transmission frameworks and the necessity to explicitly integrate animal health, milk production, handling, transport conditions, and consumption into forthcoming quantitative risk assessments of RVFV. Subsequent action must involve enhancing model accuracy and predictive capability by integrating further epidemiological predictors and revising parameter estimates based on actual data.

To address this gap, the present study aims to quantify the contribution of infected raw milk consumption by humans to the overall incidence of RVFV infection in humans, using field-collected data on climate factors and raw milk consumption practices in Kisumu, Kenya. We employ a stochastic simulation modeling approach to develop an agent-based model that simulates the transmission pathways of the RVFV pathogen in humans through raw milk consumption and vector-borne bites on farms, utilizing data and conditions specific to Kisumu, Kenya, under inter-epidemic conditions.

## 2. Materials and Methods

### 2.1. Selection of the Articles

The article selection procedure, according to question-based selection criteria, was sourced from PubMed^®^ (National Library of Medicine, 8600 Rockville Pike, Bethesda, MD 20894, USA); Web of Science^®^ (70 St. Mary Axe, London EC3A 8BF, UK); Africa Index Medicus^®^ (BP:06 Brazzaville-Congo https://indexmedicus.afro.who.int); and African Journal Online^®^ (AJOL PO Box 420 Makhanda/Grahamstown 6140 South Africa) databases. Following the elimination of duplicates, publications were incorporated through three stages: title screening, abstract review, and full-text evaluation. After the removal of duplicate articles and the exclusion of irrelevant articles by a comprehensive review of titles and abstracts, 325 articles were found and assessed for eligibility according to 27 different question categories that satisfied the inclusion criteria for this systematic review. A literature review was performed to estimate the values of parameters to be included in the study (Table 1).

### 2.2. Study Scenario and Data Source

Kisumu was purposely selected as the main study scenario due to the availability of data on RVFV prevalence and behavioral practices of populations around raw milk handling and consumption from our prior studies [64,71]. Kisumu is the largest city in Western Kenya (0.09171° S, 34.7671° E) with a population of 1,224,531 people, of whom 40% reside in informal settlements. Kisumu is 11 km south of the equator and has an average temperature of 23 °C throughout the year [72]. Meteorological data for Kisumu are composed of daily data on mean, maximum, and minimum temperature (°C) and rainfall (mm) collected from ground devices in Kenya at the study site in Kisumu from April 2014 to February 2022 [73]. Any missing temperature data were imputed using available literature and published readily by the “Visual Crossings,” which is an easy-to-use, low-cost source of historical and forecast global weather data [74].

### 2.3. Simulated Populations and Model Overview

We developed an agent-based model incorporating temperature data and conditions from Kisumu. To develop the model, we created three synthetic populations on farms: humans, cows, and vectors representing Kisumu farm dynamics. The model was configured to simulate, on a daily basis, what happens on farms when vectors, humans, and cows are present, and thus infection can occur.

To do this, we simulated 1000 farms. Each farm is initialized with a population of cattle, humans, and mosquito vectors, drawn from empirical distributions reflecting local smallholder farming conditions: herd sizes follow a binomial distribution, household sizes are drawn from a Poisson distribution anchored to a baseline of approximately four people per household with a weak positive association with herd size, and initial infection prevalence in cattle and humans is set at 2% and 1%, respectively. We assumed that new susceptible cows and humans enter the farms every 5 years [69,70,75,76]. The infectious period for RVFV was set at 7 days [77]. For prevalence of RVFV in humans, a binomial distribution with a probability of 0.01 was utilized and for cows [78], a binomial distribution with a probability of 0.02 [DAT1] was used [17].

The vector population was created using a function with the probability of the vectors doubling every 20 people up to a ceiling point, with mosquito population dynamics based on a density-dependent function and temperature, following the model developed by Mordecai and colleagues [77].

The model was configured to simulate each farm daily (2014–2022) when humans, cows, and vectors are present, and there is a potential chance of transmission if any of them is infected. The probability of a human or cow being bitten in a given farm depends on the number of mosquitoes, cows, and humans on the farm. Transmission events happen in those locations where infected vectors contact susceptible humans and cows or vice versa [79]. The mosquito population on each farm is initialized using a temperature-dependent vectorial capacity function, with the proportion of initially infected vectors derived from a steady-state approximation based on local human prevalence and ambient temperature. Mosquito bites depend on both temperature-dependent biting rate and the probability of having a successful vector–human and vector–cow encounter, which depends on temperature and the proportion of available hosts among cows and humans. The rate of biting (*a*) is temperature-dependent according to previously published work [2]. We coupled it into a binomial distribution to define the number of biting mosquitoes as follows:

*Nb*~(*Nm_t_*,*a*)

where *Nm_t_* is the mosquito population size on day *t*, *Nb* is the number of females needing to bite on day *t*, and *Nm_t_* is the size of the subpopulation of mosquitoes that day. While most models assume that all females bite when they need to, here we consider the possibility that mosquitoes cannot find blood-meal hosts at a given place and time. We assume that there is a probability for *Nb* mosquitoes to have a successful biting human-mosquito or cow-mosquito encounter (*P* (*bite*)) based on the size of *Nb* and the amount of time that humans spend in the location.

Since the primary route for vectors to be infected with RVFV is biting cows infected with RVFV, we assume the naïve mosquito population bites the infected cows to become infected. A susceptible mosquito that bites an infected cow can be moved from susceptible to exposed stage according to temperature-dependent vector competence and later be moved to infectious stage with temperature-dependent extrinsic incubation period (EIP) following functions previously described [80]. Infectious mosquitoes remain in this stage until death, for which the rate also depends on temperature using a previously published function [80].

Humans who are bitten by infected mosquitoes are moved to the infectious stage lasting seven days before moving to a recovered stage. Once infected with RVFV, the period of complete protection lasts a lifetime; therefore, the return to a susceptible state is proxied by using the number of new susceptible humans entering the farms every 5 years utilizing a random binomial distribution. Similarly, once cows are bitten by an infected mosquito, the complete protection lasts a lifetime. In the model, the susceptible cow population is replenished every 5 years as well [70,75,76] through the introduction of naïve cows to the farm. The probability that an infected mosquito bite results in human infection was set to 0.28 times the temperature-dependent transmission probability [81]. The probability that an infected mosquito bite results in cow infection was drawn from a binomial distribution with parameters set to the temperature-dependent transmission probability.

*Aedes aegypti*, although not the most extensively researched vector for RVFV, has vector competence and may play a significant role in urban or peri-urban transmission. We employed *Ae aegypti* in our model system owing to the well-established and validated climate-based characteristics and models for *Ae aegypti*. The model tracks two distinct transmission pathways on a daily timestep: vector-borne transmission, in which infected *Aedes aegypti* mosquitoes acquire RVFV from infectious cattle and transmit it to susceptible cattle and humans through temperature-dependent biting rates and host-specific transmission probabilities, and alimentary transmission, in which humans become infected through consumption of raw milk from infectious cattle, governed by a daily probability of raw milk consumption, a hypergeometric draw over the farm’s infected and susceptible cow pool, and a per-exposure transmission probability. Cattle are treated as the primary amplifying host with higher vector-to-host transmission competence, while humans are modeled as spillover hosts with a proportionally lower per-bite infection probability.

Each computational run considers a temporal window of 2866 days between 1 April 2014 and 4 February 2022, based on the availability of temperature data. Data are temporarily grouped, yielding the number of cases happening every month. At each daily timestep, new infections from both pathways (vector-borne and milk consumption) are tallied across all farms and aggregated into time series of human milk-borne infections, human vector-borne infections, and cow vector-borne infections, allowing comparison of the relative contribution of each transmission route to overall RVFV incidence across the simulation period. Results are expressed as mean and standard deviations of infections. The model and simulation were coded and run in R version 2023.06.1 + 524 (2023.06.1 + 524) (Table 2).

### 2.4. Parameter Sensitivity

To understand the transmission of RVFV through raw milk consumption, sensitivity analysis of model outputs due to variation in the probability of consuming raw milk (0.01, 0.3, 0.5, and 0.8) and the probability that infectious raw milk will cause infection in humans (0.1 and 0.4) was assessed during the simulation period. According to the literature, in Kenya, approximately 35% of people consume raw cow milk [32,83,84]; therefore, we test the sensitivity of raw milk consumption at different levels (0.01, 0.3, 0.5 and 0.8). Sensitivities were assessed on a daily timestep spanning 1000 farms.

### 2.5. Software and Code Access

The simulation model and source code are publicly available in the GitHub repository. https://github.com/atariq2891/RVFV_simulation_milk_trasnmission version 2026 GitHub, Inc., San Francisco, CA, USA (accessed on 23 July 2026). 

## 3. Results

During a period of 66 months, the model yielded a monthly mean of 0.14 (SD: 0.05) total human infections in Kisumu (milk- or vector-associated). The monthly mean of milk-acquired infections was 0.02 (SD: 0.21), and the monthly mean of vector-acquired infections was 0.12 (SD: 0.48). The monthly mean for cow infections caused by vectors was 0.33 (SD: 1.6). In general, the cow infections were twice the number of human infections (milk and vector combined) for any raw milk drinking probability over the period of 66 months.

### Potential of Raw Milk to Cause RVFV Infection in Humans

**Figure 1:**
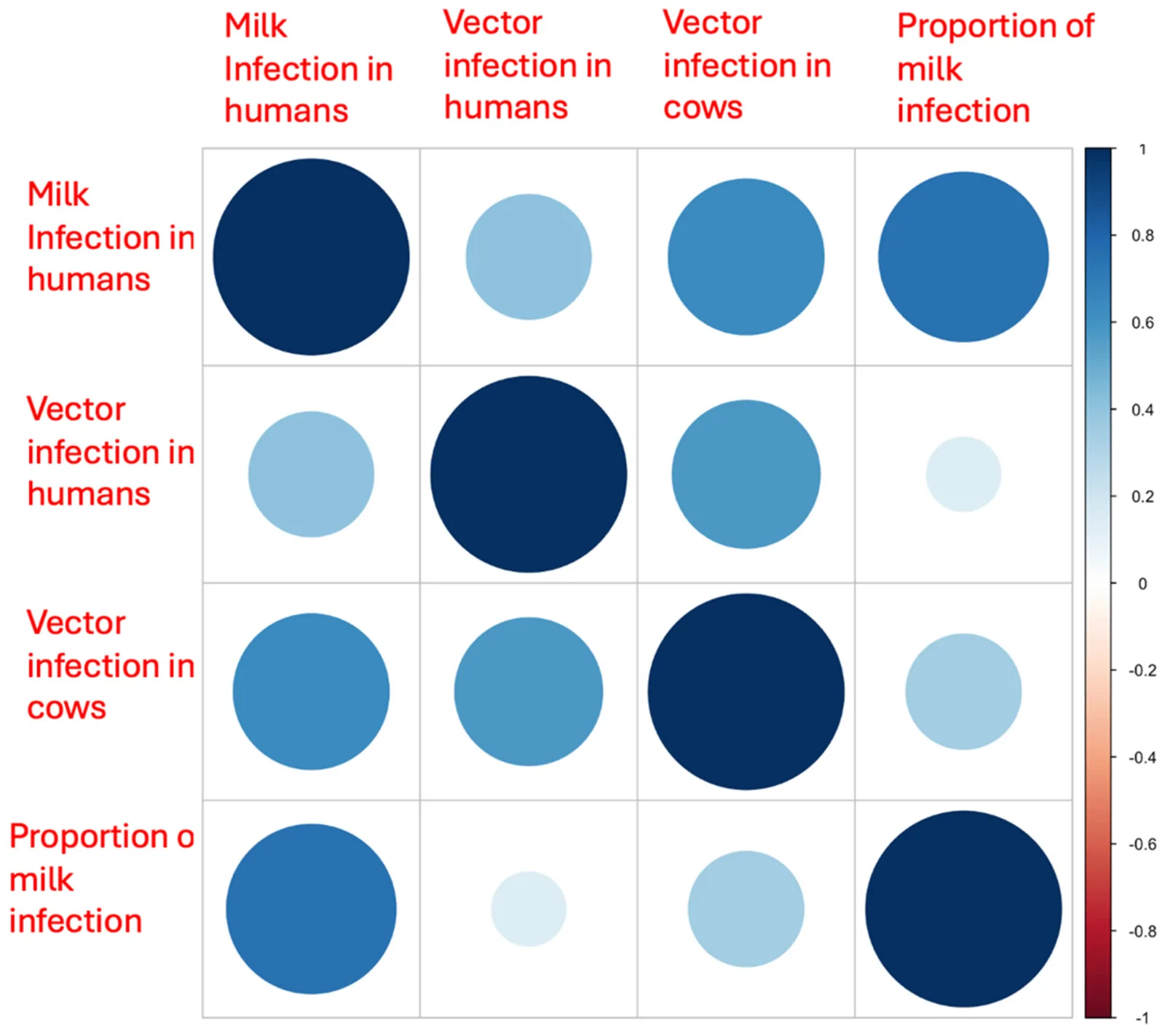
Correlation matrix showing the correlation between milk infection in humans, vector infection in humans, vector infection in cows, and the proportion of milk infection (which is the milk infection in humans divided by the sum of milk infection and vector infection in humans) when the probability of drinking raw milk is 0.3 and the probability that RVFV-infected raw milk will cause infection is 0.4. The size of the circle represents the absolute value of the corresponding correlation coefficients, and the color represents the strength of the correlation.

The correlation matrix (when the probability of drinking raw milk is 0.3 and the potential of raw milk to cause infection is also 0.4) shows positive correlations between milk-acquired infections in humans and vector-acquired infection in humans (r = 0.4), vector-acquired infection in cows and milk-acquired infection in humans (r = 0.63), and vector-acquired infection in humans and vector-acquired infection in cows (r = 0.57) (Figure 1).

To assess the potential for raw milk to transmit RVFV to humans, we varied the probability of raw milk being infectious as well as the probability of drinking raw milk.

**Figure 2:**
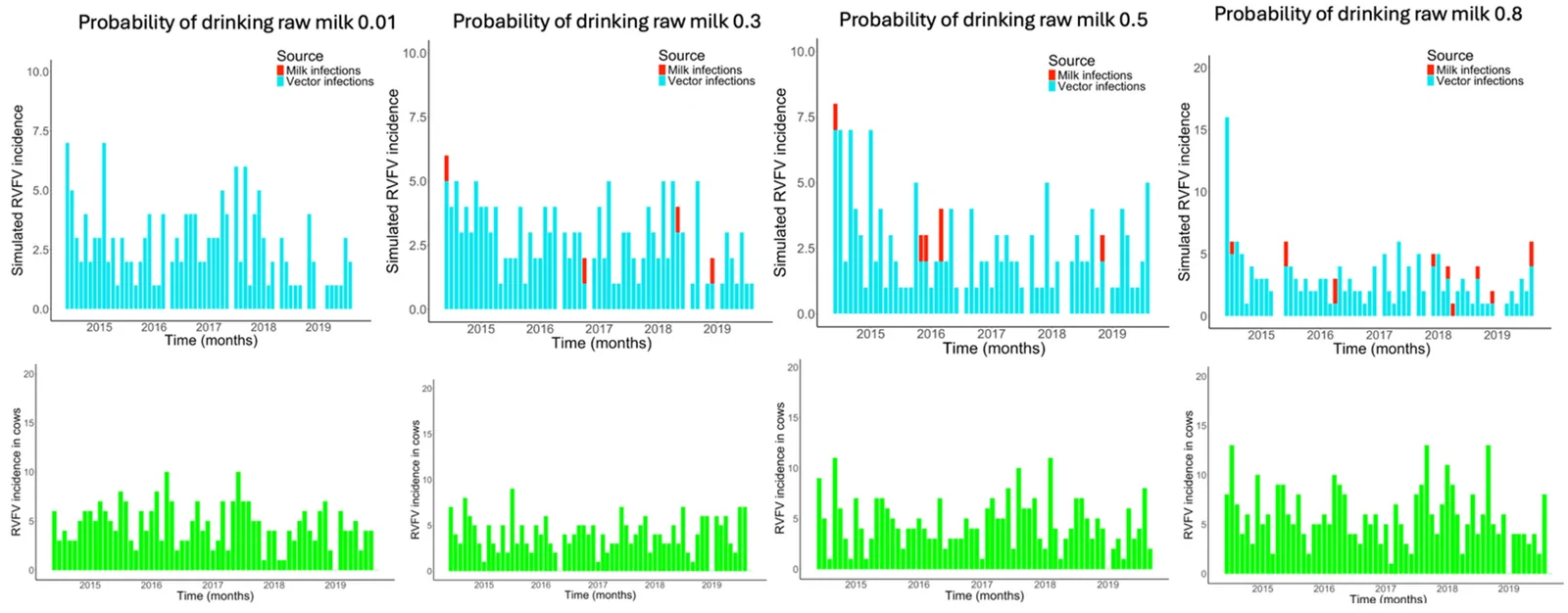
**Top panels**: Proportion of milk- and vector-acquired simulated infections in humans per month when the simulations were carried out by varying the probability of drinking raw milk from 0.01 to 0.8 and when the probability that raw milk infected with RVFV will cause infection is 0.1. **Bottom panels** show the number of simulated cow infections.

**Figure 3:**
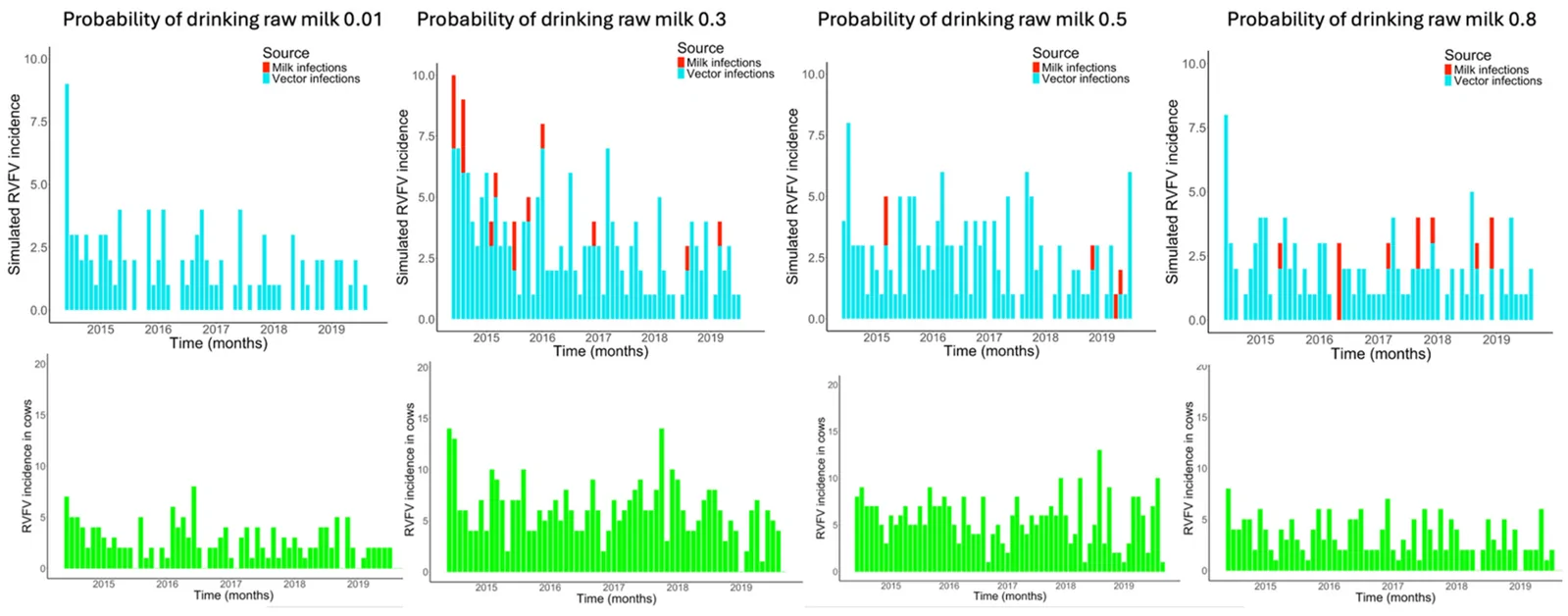
**Top panels**: Proportion of milk- and vector-acquired simulated infections in humans per month when the simulations were carried out by varying the probability of drinking raw milk from 0.01 to 0.8 and when the probability that raw milk infected with RVFV will cause infection is 0.4. **Bottom panels** show the number of simulated cow infections.

Due to a lack of information regarding RVFV transmission efficacy in milk and wide population variation in milk consumption behaviors, we chose a range of plausible values for these parameters. When the probability of raw milk being infectious is 0.1 (i.e., the probability that consumption of raw milk contaminated with RVFV will cause RVFV disease in humans is 0.1), the results of the analysis show that the percentage of cumulative RVFV infections in the human population caused by drinking raw milk at a probability of 0.01, 0.3, 0.5, and 0.8 ranges from 0.4%, 7.4%, 8.4%, and 13% respectively, of the total human infections over a period of entire simulation (Figure 2). When the simulations are run at a probability of raw milk being infectious at 0.4 (i.e., the probability that consumption of raw milk contaminated with RVFV will cause RVFV disease in humans is 0.4), we observe a greater number of infections caused by raw milk, in the range of 3–28% (Figure 3). When the probability of drinking raw milk is set at 0.01, 0.3, 0.5, and 0.8, the cumulative percentage of human infections caused by raw milk consumption is 3.7%, 21.6%, 24.6%, and 28.6%, respectively. As the infectiousness of the milk and the probability of drinking raw milk increase, the probability of raw milk consumption causing infection in the population also increases.

## 4. Discussion

RVFV is a significant threat to both human and animal health [8,17,21,22]. Our findings further suggest that raw milk consumption may be a possible but neglected route of RVFV transmission to humans in Kenya during inter-epidemic periods.

Multiple studies have linked RVFV transmission to raw milk exposure, indicating a potentially crucial area of research for future RVFV epidemics [3,32,38,85,86]. In scenarios including daily consumption of raw milk, vector-borne transmission resulted in around three times more cumulative infections over a one-year span. In accordance with previous findings, our stochastic simulations indicate that 0.4–28% of human RVFV infections may be attributed to raw milk consumption, when the probability of raw milk intake is varied between 0.01 and 0.8, and the probability of infection from drinking infected milk is varied between 0.1 and 0.4. While vector-borne transmission remains the predominant route of RVFV infection in humans in these scenarios, if RVFV is transmissible via milk consumption, this route of infection could lead to substantial numbers of infections under certain socio-cultural and ecological settings [16,41]. This aligns with serological evidence demonstrating widespread RVFV circulation in livestock, including inter-epidemic seroconversions and IgM detection, which suggest the possibility of intermittent viral presence in milk and other animal products [32,78]. Although milk is unlikely to sustain RVFV epidemics independently, it represents a secondary pathway that could contribute to human exposure, particularly in high-contact settings such as rural and pastoral communities and dairy farms [87,88,89,90,91,92].

Livestock infection dynamics are important parameters for RVFV transmission, but milk production and local distribution practices such as pooling of milk from multiple animals, transport conditions, and customer behaviors highlight the need to explicitly incorporate milk value-chain factors into future quantitative risk assessments and One Health prevention strategies [32,64,93]. Authorities recognize that early detection of livestock surveillance and epidemic prediction are effective strategies for mitigating impacts on public health and the economy during epidemics. Simultaneously, the necessary tools to guarantee proper animal health surveillance and preparedness are sometimes lacking during inter-epizootic intervals. If milk surveillance can be performed during inter-epidemic periods, it may help detect RVFV transmission early and contribute to increased outbreak mitigation and early detection [3,4].

Pathogens from human hosts, environment, dairy animals, or other species have been responsible for milk-borne diseases [89,94,95]. Various arboviral infections, such as Schmallenberg disease, tick-borne encephalitis, bluetongue disease, and Akabane, are known to be transmitted through milk [88,89,90,91,96,97,98,99]. Due to general advancements in pasteurization, public health and hygiene, and water supplies, milk-borne transmission of organisms to humans has been greatly reduced. However, RVFV has been linked to a modest risk associated with the consumption of raw milk in low- and middle-income countries [17,29,31,38,85,100,101,102,103]. In summary, these findings indicate a possible connection for the transmission of RVF infection from animals to humans in rural or pastoralist families, thus requiring the implementation of targeted community education, One Health surveillance, and preventive and control measures against the disease. This will be essential for protecting individuals from potential infection spillovers during livestock outbreak episodes. In this context, our modeling results suggest a substantial contribution of human RVFV infection from raw milk consumption (0.4–28%), which is biologically plausible, given the documented frequency of livestock infection and viral circulation at the rural and/or pastoralist population level.

There were many limitations to this study. Numerous unknown parameters exist concerning the transmission pathways of RVFV, including the virus’s latent period, the duration between exposure and infection, the varying likelihood of raw milk consumption among specific populations in Kenya, and, most importantly, the probability of the virus being infectious in raw milk through consumption. The lack of clearly defined storage and usage conditions for the milk, accompanied by uncertainties regarding viral shedding in animals, renders these estimations based on various assumptions. The infectivity rate (the percentage of individuals affected after consuming contaminated milk) remains entirely uncertain; therefore, we examined a range of possibilities aimed at exploring the raw milk consumption scenario. Multiple assumptions have been made during the stochastic building of the model. The lack of parameter estimates was a hindrance in developing a contemporary SEIR compartmental model; therefore, we developed an agent-based stochastic model. Regrettably, there are limited data regarding the alimentary route of infection in animal models or humans. Further pathogenesis studies are needed to confirm that milk consumption is sufficient for RVFV infection. Under field conditions, evaluating factors independently may not always be feasible in zoonoses with multifactorial transmission. Direct or mixed patterns of disease processes can be observed in the field. The study’s location in Kisumu, which is categorized as an urban area with fewer households involved in cattle husbandry, but where raw milk consumption remains associated with RVFV seropositivity, strengthens the plausibility of milk-acquired infections [64]. For the first time, a model of RVFV transmission via milk was simulated in this study by imitating the milk transmission model of arbovirus-borne animal diseases, including Schmallenberg disease, tick-borne encephalitis, bluetongue disease, and Akabane [87,88,89,90,91,92,97,98,104,105].

Priority future directions include conducting fieldwork to test milk for RVFV during epidemic and inter-epidemic periods. Prospective trajectories should emphasize validation of the transmission model to better quantify uncertainty around milk-borne transmission. Sensitivity analyses should prioritize parameters such as viral shedding in cattle, local raw milk consumption frequency, transportation and storage conditions of milk, and the probability of infection following ingestion. Additionally, experimental studies using animal models should further clarify parameters such as infectivity of ingested milk. Comparing our model to other models, such as stochastic, spatially explicit, or age-structured frameworks, is necessary in epidemiological contexts. RVF is most effectively managed with a One Health strategy, and these findings underscore the need for targeted One Health interventions, including routine milk testing, community-based education on boiling or pasteurization of milk, and integration of milk surveillance into existing veterinary and public health monitoring programs [3,4,5,8,43,94]. The outputs of the model can be utilized to determine the comparative effects of viable interventions, such as advocating for milk boiling, avoiding the consumption of raw milk and dairy products, implementing targeted vaccination of livestock, revising RVFV screening protocols in animals, and improving surveillance during inter-epidemic intervals. Translating these results into policy-relevant metrics may support evidence-based prioritization of RVFV control strategies within an integrated One Health approach.

## 5. Conclusions

Stochastic modeling revealed that up to 0.4–28% of human RVFV infections may be attributable to raw milk consumption during an inter-epidemic period. Vector-borne infections occur approximately five to ten times more frequently than milk-borne infections over a one-year period. While vectors remain the primary route of RVFV persistence and transmission in Kenya, milk should not be overlooked as a secondary source, but rather as a meaningful source of human infection. These results underscore the necessity of conducting a comprehensive evaluation of milk as a potential vector in terms of RVFV transmission dynamics and public health risk assessments.

## Figures and Tables

**Table 1:** Values of the parameters used in this study.

Parameter	Value
Probability of drinking raw milk	0.1–0.8 [65]
Probability that infected raw milk will cause infection	0.1, 0.4 [32,39]
Infectious period	7 days [66]
New cows and humans enter farms (proxy susceptible cows and humans)	5 years [67,68,69,70]

**Table 2:** Implementation of mosquito-related functions to model population dynamics traits.

Trait	Estimation Function				Use
	Function	c	Tmin	Tmax	
Man-biting rate (*a*)	Brière	2.02 × 10^−4^	13.35	40.08	$N_{B}\sim Bin\left(Nm,a\right)$ *
Mortality rate (*µ*)	Quadratic	−1.48 × 10^−1^	9.16	37.73	*N_D_*~*Bin*(*Nm*, *μ*) ^+^
Vector competence (*vc*)	Brière	−1.51 × 10^−3^	7.1	42.3	*E*~*Bin*(*N*_*bit*_,*vc*) ^‡^
Parasite development rate (*PDR*)	Brière	8.84 × 10^−5^	9.0	45.90	inf~Bin(E,PDR) ^¥^
Eggs per female (*EFD*)	Brière	8.16 × 10^−3^	14.7	34.4	*Eggs*~*Poisson*(*EFD*)
Mosquito development rate (*MDR*)	Brière	7.86 × 10^−5^	11.36	39.17	*r* = *MDR*∙*pEA*∙*f*(*D*) *Em*~*Bin*(*N_L_*, *r*) ^§^
Egg-to-adult survival probability (*pEA*)	Quadratic	−5.99 × 10^−3^	13.56	38.29	

Functions were extracted from previous works originally fitted by Mordecai [77,80] and later modified and used by Peña-García et al. ([82]. Functions can be either Brière [cT(T − Tmin)(Tmax − T)1/2] or quadratic [c(T − Tmax)(T − Tmin)]. * *N_B_*, the number of mosquitoes at a given location biting that specific day. ^+^ *N_D_*, the number of deaths happening in a given day at a specific location. *vc* is vector competence. ^‡^ *E*, number of exposed mosquitoes, *N_bit_* refers to those mosquitoes that bit an infected individual, so they are moved to an exposed infection status with a probability *vc*. ^¥^ *inf* refers to the moment when a mosquito is moved to the infectious stage, which is determined by a rate *PDR*. ^§^ *Em*, number of emerged mosquitoes, *N_L_* is the number of larvae at a given location.

## Data Availability

Preliminary results from this work were previously presented at the 11th Annual Stanford Global Health Convening, 2025: Wednesday, 29 January 2025, Arrillaga Alumni Center, Stanford University, Stanford, California, USA as a poster presentation and a lightning talk. https://events.stanford.edu/event/2025-global-health-research-convening#:~:text=Join%20us%20for%2011th%20Annual%20Stanford%20Global%20Health,the%20Arrillaga%20Alumni%20Center%20on%20Stanford%20University%20campus (accessed on 16 July 2026). Portions of this material were previously presented at the American Society of Tropical Medicine and Hygiene (ASTMH) 2025, Annual Meeting on 9–13 November, Metro Toronto Convention Center, Toronto, Ontario, Canada (poster presentation). The abstract could be accessed from ASTMH_2025_Annual_Meeting_Abstract_Book_56833b3855.pdf, 6000–7965, pp. 164, Abstract number 6530. https://d1lx8xdon3af7k.cloudfront.net/ASTMH_2025_Annual_Meeting_Abstract_Book_56833b3855.pdf (accessed on 16 July 2026). Portions of this material were previously presented at the 2025 Global Development Research Symposium on Friday, 25 April 2025, as a poster presentation. https://kingcenter.stanford.edu/events/student-event/2025-global-development-research-symposium?utm_source=event_email&utm_medium=email&utm_campaign=2025_research_symposium (accessed on 16 July 2026).

## Abbreviations

The following abbreviations are used in this manuscript:

RVFV	Rift Valley fever virus
RVF	Rift Valley fever
SIR	Susceptible (S), Infected (I), and Recovered (R)
SEIR	Susceptible (S), Exposed (E), Infectious (I), and Recovered (R)
EIP	Early Intervention Program
